# The Role of the Selected miRNAs as Diagnostic, Predictive and Prognostic Markers in Non-Small-Cell Lung Cancer

**DOI:** 10.3390/jpm12081227

**Published:** 2022-07-27

**Authors:** Michał Szczyrek, Paulina Bitkowska, Marta Jutrzenka, Janusz Milanowski

**Affiliations:** 1 Department of Pneumology, Oncology and Allergology, Medical University of Lublin, 20-090 Lublin, Poland; paulinabitkowska1@gmail.com (P.B.); janusz.milanowski@umlub.pl (J.M.); 2 Collegium Medicum, University of Warmia Mazury in Olsztyn, Aleja Warszawska 30, 11-041 Olsztyn, Poland; jutrzenkamarta@gmail.com

**Keywords:** NSCLC, miRNA, diagnostic marker, prognostic factor, predictive factor, therapy

## Abstract

Lung cancer remains a leading cause of cancer-related deaths worldwide, overtaking colon, breast, and prostate cancer-related deaths. Due to the limited diagnostic possibilities, it is often diagnosed after it has reached an advanced stage. The delayed diagnosis significantly worsens the patient’s prognosis. In recent years, we have observed an increased interest in the use of microRNAs (miRNAs) as diagnostic, predictive, and prognostic markers in non-small-cell lung cancer (NSCLC). The abnormal expression levels of the miRNAs could be used to detect NSCLC in its early stages while it is still asymptomatic. This could drastically improve the clinical outcome. Furthermore, some miRNAs could serve as promising predictive and prognostic factors for NSCLC. Some of the currently available studies have shown a correlation between the miRNAs’ levels and the sensitivity of tumour cells to different treatment regimens. Analysing and modulating the miRNAs’ expression could be a way to predict and improve the treatment’s outcome.

## 1. Introduction

Lung cancer is a leading cause of cancer-related deaths worldwide, with an estimate of 1.6 million caused deaths each year [1]. In the United States of America alone, 1,898,160 new cancer cases and 608,570 cancer-related deaths were projected to occur in the year 2021 [2]. The differentiation between the lung cancer forms relies on immunohistochemistry and microscopy [3]. The two main lung cancer types are: non-small-cell lung cancer (NSCLC, 85% of cases) and small-cell lung cancer (SCLC, 15% of cases) [3]. NSCLC can be further divided into two major subtypes: adenocarcinoma (AC) and squamous cell carcinoma (SCC) [4]. In the year 2015, a new tumour classification by the WHO was released. According to it, lung tumours can be divided into epithelial tumours, mesenchymal tumours, lymphohistocytic tumours, tumours of ectopic origin, and metastatic tumours. AC and SCC belong to the group of epithelial tumours [5].

Current diagnostic tools for detecting NSCLC include imaging studies (chest X-ray, CT, MRI, PET), sputum analysis, and biopsy [6]. Unfortunately, due to an initially asymptomatic tumour development, the NSCLC patients are usually diagnosed with an already advanced-stage disease [7]. Thus, the average 5-year survival is low and amounts to approximately 15% [7].

Tobacco smoke, environmental pollution, and genetics are the greatest known risk factors for NSCLC’s development [8,9]. However, it has also been reported that noncoding molecules such as miRNAs could be involved in cancer development and progression [8,9]. MiRNAs are short, endogenous RNAs that play an important role in the regulation of gene expression [10,11] and can be present in the circulating plasma [10,11]. Numerous studies have shown that specific miRNA profiles are promising cancer biomarkers, as they can be used to detect NSCLC in its early stages and predict the patients’ survival rates [7,9,11].

In this review, we aim to show the role of selected miRNAs as diagnostic, predictive, and prognostic markers in the NSCLC.

## 2. Materials and Methods

A literature review was performed using the following search engines: PubMed, ScienceDirect, and Google Scholar. For writing about the relationship between specific miRNAs and the NSCLC, we decided to exclude the articles about their roles in other cancer types when said articles did not contain any information regarding these miRNAs’ roles in the NSCLC. We found that many miRNAs had different—sometimes completely opposite—roles in various cancer types and that these roles were influenced by many factors, some of them tumour-dependent (e.g., the tumour environment). Because of that, we did not want to project data on the miRNAs’ roles in other tumour types onto their role in NSCLC. We acknowledge that the function of a selected miRNA in other cancer types could align with its already known role in NSCLC or be an indicator as to what role it might play in NSCLC. However, for the clarity and factual accuracy of this work, we decided to focus on the existing NSCLC-related data.

## 3. Results

### 3.1. Non-Small-Cell Lung Cancer

Non-small-cell lung cancer (NSCLC) is the most common type of lung cancer with a variety of subtypes: adenocarcinoma (AC), squamous cell carcinoma (SCC), and large cell carcinoma (LCC) [4]. The most frequent type of NSCLC is AC, which represents 50% of all cases [12]. It usually develops from the smaller airway epithelium and is a form prevalent in the population of never-smokers. The second most common histological NSCLC type is the SCC, which develops from the multilayer squamous lining cells [13]. Squamous cells are formed through the metaplastic change of the secretory cells and their characteristic features are keratinization and/or intercellular bridges [12]. In comparison to the usually peripherally found AC, SCC is often located in the central parts of the lung or in the main airway [13]. The least common among these lesions is the LCC, which is defined as a tumour with neither glandular nor squamous differentiation and accounts for 3–9% of all primary pulmonary lung cancer cases [14].

The early stages of NSCLC are often asymptomatic. The advanced stage of the disease may present itself with non-specific symptoms, such as a cough (seen in 55% of patients), dyspnea (45%), pain (38%), weight loss (36%), or night sweats (20%) [15].

Diagnosis and staging of NSCLC are based on imaging tests (CT scans) and histopathological reviews [16]. The treatment is stage- and type-specific, so imaging and tissue report play a key role in the selection of therapy [16,17].

### 3.2. Micro-RNA

Among the various types of noncoding RNAs, microRNAs are a class of short non-coding RNAs (built of 21–23 nucleotides) that repress the expression of about 30% of the genes at the post-transcriptional level [10,18]. They regulate the mRNA by binding their seed sequences located within their 5′ ends to the 3′-untranslated regions (3′-UTRs) of the target mRNA. This ultimately results in the mRNA’s repression through its translational inhibition and/or destabilisation [10,18]. Through that, miRNAs affect cellular inflammation, cell cycle regulation, stress response, cell differentiation, apoptosis, metastasis, migration, and hemopoiesis [10,17,19].

Currently, over 2656 mature miRNAs have been identified, many of which are disregulated or mutated in disease [20]. MiRNAs associated with carcinogenesis, malignant transformation, and metastasis are regarded as oncomirs [18,20]. Oncomirs have been extensively studied and many of them have been identified as important development regulators in numerous types of human cancers [18,20,21].

### 3.3. Micro-RNAs Could Serve as Diagnostic, Prognostic and Predictive Tools in Patients with NSCLC

**Figure 1:**
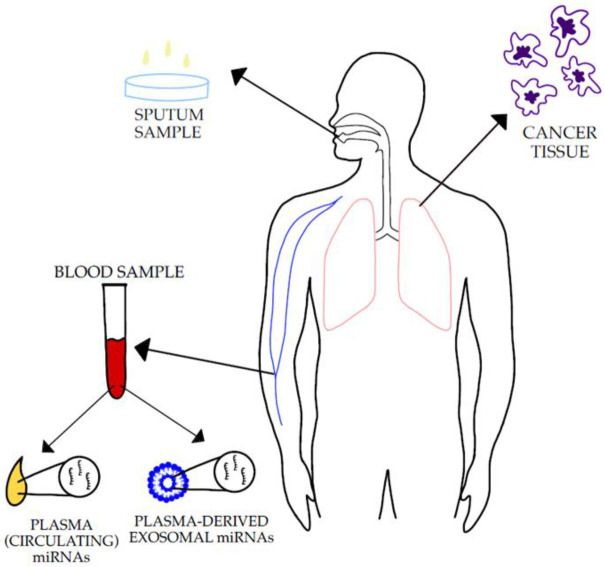
The figure illustrates the sample types in which the miRNAs’ expression levels can be measured [7,11,23,33,34,35,37].

The available data indicates that miRNAs play an important role as both tumour suppressors and oncomirs [22,23,24,25,26,27,28,29,30,31,32], and can be used as diagnostic markers [22,24,31,32,33,34,35,36]. The detection of various miRNAs in bodily fluids could serve as a non-invasive diagnostic, predictive, and prognostic tool in patients with NSCLC (Figure 1) [7,33,35,37].

### 3.4. The Overexpression of Some miRNAs Could Contribute to the NSCLC Development and Increase Tumour’s Resistance to Certain Treatment Methods

MiR-20 was upregulated in the NSCLC patients compared to the patients with other lung diseases [38]. Its levels correlated with the TGF-beta and VEGF expression [38]. The data on its role in cancer progression is partially contradictory and the discrepancies could be a result of different research methods and subject selection criteria [38].

MiR-21 was found in various cancer types, including lung cancer [24], in which it acted as an oncomir [39,40]. It has been shown that the miR-21 was connected to the increased proliferation, invasion, and metastaticity of the tumour cells, as well as to their decreased apoptosis [24,39,41] and increased angiogenesis [40,41]. The miR-21 expression was found to be increased both in the plasma and in the tissues of the NSCLC patients when compared to the healthy control group [40]. The changes in its expression between stage I and stage II AC in a study conducted by Landi et al. indicated that miR-21 could be a good tumour progression monitoring tool [39]. Its plasma levels were also shown to drastically differ between the pre-operation and post-operation patients [7]. Its high expression was associated with poor prognosis [28].

MiR-21 was expressed more in AC than in the SCC [39]. It has been found that the high miR-21 levels in stage II AC allow it to be differentiated from the SCC. However, this did not apply to the stage I tumours [39]. Additionally, the miR-21’s expression rose proportionally with the number of cigarettes smoked per day in the SCC patients, but not in the AC patients, which indicated the existence of a histology-specific response to the tobacco-related carcinogens [39].

It has been shown that miR-21 expression is negatively regulated by PD-1 and could be increased through PD-1 inhibition [22]. Furthermore, Leonetti et al. found, that miR-21, as well as miR-27a and miR-181, could have a prognostic and predictive value in patients treated with EGFR-TKIs ((TKI—tyrosine kinase inhibitor, EGFR—epidermal growth factor receptor) [42]. This data indicates that miR-21 might be a useful diagnostic, predictive, and prognostic factor [39], as well as a potential treatment target (Table 1) [22].

It has been discovered that the serum miR-21-5p is particularly upregulated in stage I and II NSCLC patients [34]. The plasma-derived exosomal miR-21-5 levels in the NSCLC were different than in the healthy control group samples but not significantly changed in comparison to BLL (benign lung lesion) samples [34]. In addition, no significant correlation was established between the miR-21-5p levels in the plasma-derived exosomes and tumour-derived exosomes [7].

MiR-25 expression was increased in tissue [43,44] and plasma samples obtained from the NSCLC patients [43,45]. It was higher in advanced-stage tumours than in the early-stage ones [44]. Furthermore, its expression correlated with the radiosensitivity of the NSCLC [43]. MiR-25-3p was also found to be overexpressed in cisplatin-resistant NSCLC [46] (Table 2).

MiR-26a was found to have an oncogenic potential [39,48]. Its expression was increased in tissue samples of both AC and SCC and it differed largely in the histological samples of these tumours in stage I and stage II cases [39]. However, no significant difference was observed in histological samples in advanced stages [39]. Since miR-26a was higher in the AC tissue samples, it could be more relevant to its development than to the SCC tumour formation [39]. An increased miR-26a expression was linked to cisplatin resistance [48,49] (Table 2), at the same time, however, it was shown to possibly have an anti-tumour effect in patients with docetaxel-resistant lung AC [48]. In their study, Monastirioti et al. found that the higher circulating miR-26a levels were a negative prognostic factor in SCC patients [49], but so was the lower tissue miR-26a expression in both SCC and non-SCC NSCLC patients [49].

The high miR-31 expression was also associated with worse NSCLC prognosis [28]. MiR-31-5p was found to inhibit cisplatin-induced apoptosis and to possibly promote tumour cell invasion in NSCLC [28] (Table 2).

The role of miR-141’s overexpression varied depending on the sources—some deemed it to be a pro-angiogenetic factor, some pointed towards it having anti-angiogenic properties [40]. In a study conducted by Wu et al., the plasma miR-141-3p was strongly upregulated in stage I and II NSCLC patients, while the plasma-derived exosomal miR-141-3p’s expression did not differ from the healthy patients’ and benign lung lesion patients’ values [34].

MiR-155 plays an important role in the immune system’s activation and can promote tumour immune escape and its growth by inhibiting the tumour-related immune cells. It has been shown to induce apoptosis in CD8 T-cells and inhibit their function [22]. It also targets the anti-PD-1 and anti-PD-L1 antibodies. It has also been shown to enhance PD-L1 expression in lymphoma cells [22]. MiR-155 was overexpressed in NSCLC and, according to some sources, its high expression correlated with poor overall survival in lung AC and lung SCC patients [40]. However, other studies found it to have no prognostic impact, although one of them did find a correlation between the miR-155 expression and the histological tumour subtype [40]. According to another study, the plasma-derived exosomal miR-155-5p level was lower in NSCLC patients than in healthy subjects, but not compared to patients with benign lesions, and plasma miR-155-5p expression was similar in all groups [34]. It has also been speculated that the sputum miRNA analysis could serve as a tool to diagnose NSCLC based on the contained miR-155 and miR-22 levels [7].

MiR-182 belongs to the miR-183 family [55]. It was found to be often upregulated in cancers [40], including NSCLC [56], and thus it could be considered as an oncomir [40,55,56]. However, its role must be further investigated since it has been shown to have various antagonising effects [40]. It has also been found that miR-182’s expression in the plasma-derived exosomes correlated with its tissue levels in stage I NSCLC [7] and that the circulating miR-182’s expression was higher in NSCLC patients than in the healthy control group [55]. It was also upregulated in HPV-DNA positive NSCLC patients in comparison to the HPV-DNA negative ones [55]. Gao et al. [56] demonstrated that miR-182-5p was upregulated in NSCLC tissues in comparison to non-cancer lung tissues and that it played a role as an oncogene in NSCLC. This means it could be used to differentiate NSCLC from non-cancerous lung tissues [56]. Its expression was found to be insignificant to the survival outcome of NSCLC patients [56].

Another member of the miR-183 family is miR-183, whose serum expression was increased in cases of NSCLC, when compared to a healthy control group [55]. Wu et al. demonstrated that the circulating miR-183 levels were higher in the HPV-DNA positive NSCLC patients in comparison to the HPV-DNA negative ones [55]. It has also been speculated that miR-183 could be related to the NSCLC metastasis [55].

MiR-191’s expression was increased in the serum of NSCLC patients [57] and in NSCLC tissue samples [58] and was found to act as an oncomir [58]. Its expression was correlated with the prognosis [7,39]. Its upregulation in smoking male patients with SCC was also shown to be associated with worse survival [39]. However, its lower expression has also been linked to worse survival [39] and to an increased radiation resistance [59].

MiR-210, a proangiogenic miRNA, was found to be overexpressed in the serum exosomes obtained from untreated NSCLC patients [40]. It has also been shown that miR-210’s expression in the plasma-derived exosomes was similar to its expression in the stage I lung cancer tissues [7]. Furthermore, Wu et al. reported that the levels of the circulating miR-210 were higher in NSCLC patients than in the healthy control group [55]. Additionally, the circulating miR-210’s expression was higher in HPV-positive NSCLC patients than in HPV-negative NSCLC patients [55].

The miR-221/222 cluster was found to be overexpressed in the NSCLC [40,60]. MiR-221’s increased expression has been linked to a worse prognosis in lung cancer patients [60] and could serve as a prognostic tool [7]. Plasma miR-222-3p’s expression was found to be particularly upregulated in the NSCLC patients in comparison to healthy subjects but not in comparison to subjects with other lung diseases [34]. However, no difference was noticed between the plasma-derived exosomal miR-222-3p’s expression in NSCLC patients and the one in healthy subjects [34].

According to a study by di Paolo et al., miR-221-3p inhibition combined with miR-126-3p augmentation induced tumour apoptosis [60]. This method was also well tolerated by normal cells [60].

MiR-486 expression in plasma-derived exosomes was similar to its level in the stage one lung cancer patients’ tissues [7]. The plasma-derived exosomal miR-486-5p levels were notably higher in NSCLC-samples than in both patients with benign lung lesions and healthy subjects [34]. On the other hand, Wu et al. found that the expression of the circulating miR-486-1 and miR-486-2 was decreased in the NSCLC patients [55].

MiR-494 expression is upregulated in tumour tissues in NSCLC [52]. It has been determined that miR-494 promotes angiogenesis in the A549 NSCLC cell lines and that hypoxia could cause an increase in its expression [40]. Its higher levels were also associated with worse prognosis and increased cisplatin resistance [52] (Table 2).

An increase in miR-556-5p’s expression was shown to correlate with the prevalence of cisplatin resistance in the NSCLC patients’ tissues and cells [46]. It has been demonstrated that the miR-556-5p knock-down had an anti-tumorigenic effect and promoted cisplatin sensitivity [53] (Table 2).

Zhou et al. found that the combined serum levels of miR-601 and miR-942 could be used for early tumour detection and prognosis prediction in NSCLC cases [61]. Both of those were increased in samples derived from the NSCLC patients in comparison to the healthy control group [61]. Their overexpression was connected to poor prognosis and worse clinical outcome [61].

MiR-629 serum expression was significantly increased in the NSCLC patients compared to patients with benign lung diseases and healthy subjects [62]. Its expression was also shown to be upregulated in the NSCLC tissues and cells [63]. Based on the AUC (area under the ROC curve) values, it had a better diagnostic potential than the CEA and CYFRA 21-1 [62]. It has been observed that the higher miR-629 levels correlated with worse disease-free survival and overall survival [62]. The miR-629 expression was decreased in patients after receiving surgical treatment [62]. Its increase was correlated with a worse prognosis [63].

MiR-15 was identified as an oncomir in various cancer types, including the NSCLC [47]. MiR-15b overexpression has been linked to cisplatin resistance. Anti-miR-15b notably restored cisplatin sensitivity in NSCLC cells [47] (Table 2). The miR-15-16 cluster’s expression was limited by hypoxia, which supported tumour angiogenesis and metastasis [40]. MiR-16 was found to be downregulated in NSCLC and other tumours [64]. Navarro et al. reported a correlation between poor prognosis and alterations in miR-16 level—both a decrease and an increase. They found that a high miR-16 expression was a marker for disease-free survival and that both high and low miR-16 levels were markers significant to overall survival [64].

MiR-23a was found to promote the lung tumour’s angiogenesis in normal and hypoxic environments [40]. The miR-23a-3p levels in the circulating exosomes correlated with those from tissue samples, however, no correlation was found between the plasma miRNA content and the exosomal miRNA content [7].

Song et al. found that miR-4443 expression was higher in tissue-derived exosomes of the NSCLC patients with cisplatin-resistance, compared to those derived from NSCLC patients who responded to the treatment. They established that miR-4443 promoted cisplatin-resistance and tumour growth [54] (Table 2). It has also been found that high miR-4443 expression promotes the epirubicin resistance of the NSCLC cells [65].

### 3.5. A Decrease in Expression of Some miRNAs Could Contribute to the NSCLC Development and Increase Tumour’s Resistance to Certain Treatment Methods

MiR-9 could play a role of an oncomir in lung cancer [66] and be a poor prognostic marker in the NSCLC patients [32], however, the data on its role in the tumour development was partially contradicting and inconclusive [66]. Sromek et al. found that the plasma miR-9 levels in untreated SCC patients were not differing from the healthy subjects’ values, but they were significantly lower in the AC patients [67]. According to Chen et al., its expression could be downregulated by erlotinib [66], which would make it a potential therapeutic target. The miR-9 overexpression was also shown to inhibit the effects of erlotinib on the tumour [66].

Poor expression of miR-29a in the SCC patients showed its important role as a tumour suppressor and its relevance in tumour development [39]. The MiR-29a levels allowed for the differentiation between the AC and the SCC [39]. MiR-29b was also determined to be a tumour cell proliferation, migration, invasion [68], and metastasis suppressor [40]. On the other hand, Zhou et al. found that the serum miR-29c-5p expression was slightly higher in the NSCLC patients than in the healthy subjects [61].

MiR-30a expression was lower in the NSCLC tissues than in the adjacent normal lung tissues. It also rose notably after neoadjuvant chemotherapy [69]. MiR-30a-5p was shown to have tumour-suppressive properties [28] and was under-expressed in lung AC and lung SCC [70]. Jiang et al. demonstrated in vitro that a lower miRNA-30a-5p expression in the lung AC cases could be associated with worse clinical outcomes [70]. Low miR-30a expression was more common in large tumours with lymph node metastasis and advanced TNM stage [69], whereas patients with an increased miR-30a expression had better prognosis [28,69] and 5-year survival [69]. It has been documented that the miR-30a/Beclin 1 axis promoted chemosensitivity in NSCLC [69].

MiR-33a can inhibit lung tumour growth [71]. The expression of miR-33a has been shown to negatively correlate with PD-1, PD-L1, and CTLA4 expression [22,23]. Increased miR-33 levels and decreased PD-1 expression were linked to a better prognosis [23].

The miR-34 family members were shown to inhibit cell migration, invasion, proliferation, and survival. They also reduce the tumour cells’ EMT (epithelial-mesenchymal transition), stemness, and drug resistance [5]. The miR-34a and miR-34c expression was particularly high in AC tissue and correlated with the prognosis [7]. MiR-34a and miR-34c-5p levels could also be used to predict survival in the early-stage SCC smoking male patients—their lower expression was associated with poor survival [39]. Garinet et al. demonstrated that the miR-34b and miR-34c presence in the tumour samples was linked to a better prognosis and lower relapse risk in NSCLC patients with a high epithelial mesenchymal transition score and low miR-200 levels [29].

It has been shown that miR-34a delivery combined with radiation therapy led to a synergistic effect and induced a tumour response via PD-L1 targeting and thus increasing the CD8 T-cells’ tumour infiltration [22]. It has also been reported that the overexpression of the miR-34b and miR-34c downregulates the PD-L1 expression [22].

In their study, Xu et al. discovered that miR-99b expression was low in the NSCLC tissues and cells. They demonstrated the potential of miR-99b to inhibit cell invasion and migration in NSCLC cases [72].

MiR-126 could be used as a biomarker in NSCLC diagnosis [7] and treatment [73]. Its expression in plasma-derived exosomes was found to be correlated with its expression in the tumour tissues of stage I lung cancer patients [7]. An increase in miR-126 expression has been linked to an increased overall survival from NSCLC [28,74]. MiR-126-3p is known to be a tumour suppressor [28,41] and a decrease in its expression has been noted in multiple tumour types [41]. It has been established that it could serve as a diagnostic factor due to a significant decrease in its expression in advanced-stage NSCLC compared to the early-stage NSCLC cases [41]. It was found to be strongly downregulated in the NSCLC [40] cell lines [27,75,76], tissues [27,74,75,76], body fluids [74], and serum-derived exosomes [73]. An increase in its expression supresses the aggressiveness of the tumour cells through various pathways [27,40,74,75,77]. The overexpression of the bone marrow-derived exosomal miR-126-3p was found to suppress the development of NSCLC through the downregulation of tyrosine-protein phosphatase non-receptor type 9 [78]. However, it is important to acknowledge that another study found that the plasma-derived exosomal miR-126-3p was downregulated in comparison to healthy subjects, but not in comparison to BLL patients [34]. Moreover, miR-126-5p expression was found to be lower in NSCLC tissues compared to the surrounding tissues, as well as in cisplatin-resistant tissues compared to the cisplatin-sensitive tissues [50]. It has been established that miR-126-5p increases the cisplatin-sensitivity of cancer cells, including cisplatin-resistant cells [50] (Table 2). Furthermore, the research performed on mouse models indicated that miR-126’s delivery with the use of lung-specific exosomes derived from breast cancer cells could be a potential therapeutic method in patients with NSCLC [79].

MiR-146 has tumour-suppressive properties [25]. It targets the EGFR gene, which can be mutated in patients with lung AC [25]. Its expression could be increased by cryptotanshinone [25]. MiR-146a’s expression positively correlated with EGFR TKIs’ and cetuximab’s therapeutic activity in subjects with NSCLC. It also improved the cancer cells’ cisplatin-sensitivity, and its lower expression was associated with an increase in cisplatin-resistance in NSCLC [25] (Table 2). The plasma miR-146a-5p was expressed stronger in patients with stage I and II NSCLC than in healthy volunteers, but not stronger than in it was in patients with benign lung lesions [34]. Its expression in plasma-derived exosomes was notably higher in the NSCLC-samples than in the other two groups [34].

MiR-154’s expression was lower in the NSCLC tissues than in healthy lung tissues [80]. In vitro, the increased miR-154 expression was shown to inhibit NSCLC cells’ migration and invasion [62]. The miR-154 expression was independent from gender and age. Its decrease, however, was strongly associated with a larger tumour size, advanced TNM stage, and metastasis presence [80]. The miR-154 transfection inhibited tumour growth [80].

In a study conducted by Zhou et al., miR-129-2 expression was reduced in the NSCLC tissues and cell lines in comparison to the adjacent tissues. The researchers found that increased miR-129-2 expression promoted chemosensitivity and induced cell apoptosis in NSCLC [81]. MiR-129-5 was found to improve the treatment outcomes in the NSCLC patients by promoting the tumour cells’ radiosensitivity [82]. It was also shown to be under-expressed in the NSCLC tissues in comparison to the adjacent normal tissues [83]. Its increased expression in the tumour tissues was linked to a decreased proliferation and increased apoptosis in the tumour cells [83]. It was also shown that its upregulation limited the migration and invasive activity of the tumour cells [84].

The miR-200 family has been established to play an important role in the suppression of the epithelial-mesenchymal transition [85]. Furthermore, it has been established that miR-200c’s increased expression enhances the radiosensitivity of the A549 cells [86]. MiR-200b in physiological amounts inhibits angiogenesis. Its downregulation may be stimulated by hypoxia and is followed by increased angiogenesis [40]. In our previous study, we demonstrated that patients with proportionally higher tissue miR-200b expression responded better to the immunotherapy—specifically to treatment with nivolumab or pembrolizumab. They also obtained better progression-free survival values [87]. The expression of both miR-200b and miR-200c was negatively correlated with the expression of the PD-L1 protein on tumour cells [87].

MiR-206’s expression was decreased in NSCLC [88,89]. It was shown to inhibit angiogenesis, proliferation, migration, invasion [40], and metastasis [88] in NSCLC.

MiR-375 was shown to have a tumour suppressive effect [90,91]. Its expression was found to be significantly decreased in NSCLC patients’ plasma [90,92] and in NSCLC cells [91,93]. The miR-375 downregulation coincided with a worse prognosis [93]. Its upregulation could prevent the tumour growth and promote the tumour cells’ apoptosis [91].

Hashemi et al. found that miR-377 expression was significantly lower in human NSCLC tissues and cell lines in comparison to the non-tumour tissue samples, and they also reported that miR-377 over-expression reduced the cancer cells’ proliferation and enhanced their apoptosis, thus making it a potential therapeutic target [94].

MiR-451 was found to have tumour growth inhibiting properties [95] MiR-451a was identified as a cell migration and invasion suppressor in NSCLC [28]. Its expression was decreased in the serum of the NSCLC patients [55] and in the NSCLC tissues [96]. Its increase was linked to a longer survival [55], while lower values correlated with a poor prognosis [96]. However, the data on its prognostic value is not fully conclusive [28].

Enhanced miR-497 expression was shown to inhibit angiogenesis, growth, and invasion of NSCLC cells, whereas its decrease promoted cell invasion and tumour growth [40].

MiR-567 expression was found to be lower in the NSCLC tissues compared to the adjacent normal tissues. Its decrease was connected to poorer prognosis, when compared to subjects with higher miR-567 expression. The miR-567 overexpression was also shown to inhibit the NSCLC cells’ proliferation. Moreover, it has been stipulated that miR-567 could supress malignant tumour progression in NSCLC patients by regulating cyclin-dependent kinase 2 (CDK2) [97].

In vitro, increased miR-638 expression inhibited the invasive abilities of NSCLC cells [98]. An increase in its expression after chemotherapy treatment was linked to an increased survival rate among the NSCLC patients [26]. A decrease in miR-638 levels has been shown to promote the tumour cell’s development and their EMT (epithelial to mesenchymal transition) [26]. An inverse correlation between the miR-638 levels and the lymph node metastasis rate was observed [26]. Thus, its levels could serve as a prognostic factor.

According to some studies, higher expression of miR-708 was associated with longer survival [29]. Some researchers speculated that miR-708-5p upregulation promotes tumour growth and NSCLC cell invasion [28,99]. However, others found that miR-708-5p was decreased in metastatic lung cancer tissues and cells [28,99]. Additionally, Monteleone et al. observed that miR-708-5p enhanced the effectiveness of erlotinib and paclitaxel [100].

Let-7 is a family of miRNAs, which was initially discovered in Caenorhabditis elegans [101,102]. In humans it was shown to control the stem-cell division and differentiation [101] and supress the tumour development through various pathways [39,103,104]. It has been demonstrated that the let-7 miRNAs could be used as predictive and prognostic markers in patients with lung cancer [7,25]. Their decreased expression has been linked to the development of aggressive cancers [102], worse prognosis [31,39], and poor post-operative survival [39]. Landi et al. observed that the let-7 miRNAs’ expression levels were much lower in the SCC than in the AC stage I and II patients [39].

Let-7a-5p was shown to have suppressive properties against lung cancer and to be under-expressed in NSCLC [90]. Let-7b levels were lower in tumour tissue compared to non-tumour tissue derived from NSCLC patients [40]. Subjects with decreased let-7b expression had shorter progression-free survival and overall survival [40]. At the same time, let-7b-5p’s and let-7e-5p’s expression levels in the plasma-derived and tissue-derived exosomes were found to correlate with each other, but not with the serum levels [7]. The lower let-7e expression was associated with poor survival [39]. Wang et al. found that the let-7f-5p expression was decreased in the plasma-derived exosomes of the NSCLC patients of all stages, compared to the healthy subjects [31]. However, they also reported that higher levels of let-7f were reported in patients with more malignant tumours [31]. Multiple studies demonstrated the tumour suppressive properties of the miR-202, and a connection between its decreased expression and tumour development [103]. On the other hand, Monastirioti et al. observed that increased serum miR-202 expression correlated with poor survival in patients with advanced stage NSCLC [49]. At the same time, a KM plotter analysis, which was performed on samples retrieved mainly from patients in early disease stages, pointed towards a correlation between lower tissue miR-202 expression levels and worse prognosis [49]. Zhou et al. have demonstrated that miR-98 expression was depleted in the tumour tissues, compared to the adjacent tissues [105]. Its increased expression was related to better overall survival (Table 3) [105].

In female AC subjects, an inverse correlation was reported between smoking and the let-7 family’s expression [39]. At the same time, no relationship between smoking and miRNAs expression was reported in male AC patients, as well as both male and female SCC subjects [39].

Wu et al. demonstrated that miR-4782-3p inhibited cell proliferation in NSCLC and identified the increased miR-4782-3p levels as a positive prognostic factor in patients with NSCLC [106].

Wang et al. found that miR-320a and miR-622 expression was decreased in the plasma-derived exosomes of the NSCLC patients, compared to the healthy subjects [31], and that they had a good diagnostic ability for metastasis [31]. It has also been shown that miR-320 expression was lower in the NSCLC tissues in comparison to healthy adjacent tissues [107]. Additionally, a decrease in miR-320’s expression was linked to cisplatin resistance (Table 2) and ionizing radiation resistance in the NSCLC patients [51]. Their diagnostic value of these miRNAs was especially high in combination with CEA and Cyfra21-1 [31]. Wang et al. stipulated that miR-320a and miR-622 played an oncogenic role in the NSCLC cases [31], however, further investigation of these miRNAs is necessary, especially since other sources assign the miR-320 family members to the group of tumour suppressors [31,51,107].

It has been demonstrated that alterations in the levels of certain miR-17 family members occur in lung cancer [108,109,110]. MiR-17-5p expression was lower in NSCLC patients with erlotinib resistance [109]. On the other hand, its increase was shown to potentially promote gefitinib resistance [110]. However, the currently available data is strongly contradictory and thus inconclusive as to what exact role it plays in NSCLC development [108]. Migdalska-Sek et al. demonstrated that the miR-17’s expression differed depending on the tumour’s histopathological subtype and stipulated that it could serve as a diagnostic tool in NSCLC [108].

Chaniad et al. proposed the use of miR-20a and miR-223 in lung cancer therapy due to their modulating effects on some of the tumour promoting cytokines [38]. MiR-223 expression correlated with the TGF-beta levels and negatively correlated with the VEGF concentration [38]. Plasma and plasma-derived exosomal miR-223-3p’s levels were higher in stage I and II NSCLC patients in comparison to the healthy volunteers, but not in comparison to patients with benign lung lesions [34].

## 4. Discussion

**Figure 2:**
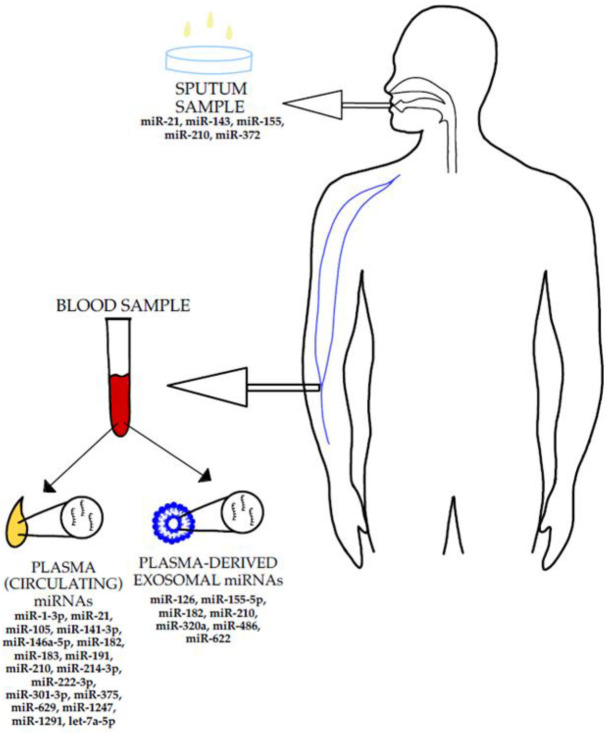
The figure illustrates the sample types that could be utilised to examine the miRNAs’ expression levels in a non-invasive manner [7,31,34,55,62,90,111,112].

Many miRNAs could potentially be used as non-invasive diagnostic tools in NSCLC. The currently available literature proposes a variety of solutions. Roa et al. selected five miRNAs in order to create a diagnostic panel for sputum testing: miR-21, miR-143, miR-155, miR-210, and miR-372. The panel’s sensitivity in detection of the NSCLC was established to be 83,3%, with a 100% specificity [111]. Ying et al. proposed the use of five serum miRNAs for a NSCLC diagnostic panel, whose diagnostic accuracy they concluded to be higher than the CEA’s accuracy, with its sensitivity being 83% and its specificity being 91%. The utilised miRNAs were as follows: let-7a-5p, miR-375, miR-1-3p, miR-1291, and miR-214-3p. The first two were under-expressed in lung cancer, the latter three were overexpressed in comparison to healthy subjects [90]. Dong et al. determined that miR-105, miR-1247, and miR-301-3p were overexpressed in the early-stage NSCLC patients’ plasma in comparison with healthy subjects [112] and that they had an especially good diagnostic value when combined with CEA [112]. The obtained data demonstrates that there are various potentially effective ways of non-invasive NSCLC detection (Figure 2), which could be implemented as a method of NSCLC-screening in patients with risk factors. This would improve the detection of early-stage NSCLC, which has been linked to better patient survival [113]. However, to confirm the efficiency of these methods, further research in this direction would be required.

According to some studies, miRNAs could be utilised to not only detect NSCLC overall but also to differentiate between the different histological tumour types—especially between AC and SCC [7,39].

The HPV-16 was found in lung cancer tissues and has been suspected to be one of the NSCLC causes [114]. Research indicates that the circulating miR-144, miR-182, and miR-183 could allow for the differentiation between the HPV-DNA-positive cases and the HPV-DNA-negative ones [55].

MiRNAs could also allow us to predict survival, assess the efficacy of selected treatment methods in single patients, and adjust the treatment strategy accordingly. Furthermore, the modulation of their expression levels could be a way to increase the treatment’s efficiency.

MiR-21 [22], miR-33a [22,23], miR-34a, miR-34b, and miR34c [22] affect the immune checkpoints. The modulation of their expression could be a way to improve the anti-tumour response. In our previous study, increased miR-200b expression as well as decreased miR-429 and miR-508-3p expressions were associated with better clinical outcomes in patients receiving immune checkpoint blockers [87]. However, further research in this direction is required.

The following four miRNAs were significantly upregulated in the plasma-derived exosomes of the NSCLC patients with osmertinib resistance: miR-1468-3p, miR-323-3p, miR-5189-5p, and miR-6513-5p [115]. Measuring their levels could not only allow for a more accurate prediction of the treatment response, but also be used to screen the osmertinib-treatment candidates for osmertinib resistance. Its early detection would allow the physicians to consider alternative and potentially more effective treatment methods. It would be a way to not only improve the patients’ prognosis by creating a more personalised and effective treatment plan, but also limit the health care system’s expenses on ineffective treatment methods. Higher miR-17-5p and miR-29a and lower let-7b expression could promote gefitinib resistance [110], which makes them potential targets in the gefitinib-resistance treatment.

MiR-146 could be used to estimate the therapeutic efficiency of cetuximab [25]. Mir-25 [43], MiR-191 [59], and miR-200c [86] could be potential targets in regulating the tumour’s radiosensitivity by adjusting their expression levels in NSCLC patients. Mir-30a [69] and miR-638 [26] expression could be measured in order to monitor the efficiency of the chemotherapy. Regulating the miR-129-2 levels, on the other hand, could be a way to enhance the chemotherapy’s effectiveness [81]. The overexpression of the following miRNAs was shown to contribute to the cisplatin-resistance of the NSCLC cells: miR-15 [47], miR-25 [46], mir-26a [48,49], miR-31 [28], miR-494 [52], mir-556-5p [53], miR-4443 [54]. On the other hand, the increased expression of the miR-126-5p [50], miR-146a [25] and mir-320 [51] improved the cells’ cisplatin sensitivity. These miRNAs’ levels could be measured to assess the potential effectiveness of the cisplatin treatment. Alternatively, their modulation could be used to limit the cisplatin resistance and to increase the tumour’s response to the treatment. A prolonged exposition of the NSCLC cells to cisplatin could result in them acquiring a cisplatin-resistance [47]. The detection of miRNAs could be a way to monitor the cisplatin-sensitivity of the patients treated with it and to detect and counteract the rising cisplatin-resistance.

MiR-21, miR-27a, miR-181 [42], and miR-146a [25] could be used as prognostic markers in patients treated with TKIs [34,42]. Xia et al. demonstrated the potential role of serum-derived exosomal miR-1169 and miR-260 in differentiation between the NSCLC patients with a mutant EGFR and those with the wild-type EGFR [36]. This would allow the prediction of the effectiveness of the EGFR-TKI treatment, which was shown to have good effects in patients with mutant EGFR, but poor efficiency in patients with the wild-type EGFR [36]. However, due to a low amount of available data, both of these miRNAs need to be further researched in order to confirm this application and better understand their overall role in the tumour-development process.

The serum miR-24 levels in pre-operative patients were found to drastically differ from those in post-operative patients [7] and thus could be used to monitor the patients’ response to treatment.

The miR-25 [44], miR-30a [69], miR-126 [41], and miR-154 [80] expression changes varied between the early and advanced stage NSCLC and thus could be used to monitor the disease progression.

The miRNAs seem to be a very promising diagnostic tool, prognostic marker, and also a potential treatment target. Despite a large amount of available sources and some very promising results, to this point, there are many gaps in knowledge that need to be filled.

Some studies reported that the differences between the patients of male sex and female sex could contribute to different miRNA expression levels, and potentially be explained by hormonal differences [39]. This would mean that patients with atypical hormonal status—for example, due to a hormonal imbalance or receiving hormonal therapy—could have strongly altered miRNA levels in comparison with the subjects that were enrolled in these studies. This could worsen the miRNAs’ diagnostic capabilities in those groups and lead to false results. The therapeutic effects of the miRNAs could be altered by hormonal alterations as well. Further research in this direction is required with consideration of various hormonal changes and interventions.

Additionally, the miRNAs’ expression levels could depend on the subjects’ age. For example, miR-21 expression was shown to be inversely correlated with it [42].

Multiple studies have shown that the miRNA expression differs depending on the disease progression. Certain miRNAs’ levels were only altered in patients with a certain illness stage [31,34,90]. For example, Ying et al. found that miR-361-5p’s expression was increased only in stage I and II stage NSCLC [90]. Furthermore, a lot of the research we found did not utilise all possible forms of tissue samples [7,34,39,49]. This is especially important since more than one study reported that the miRNAs’ expression levels in different sample types were not always correlated [7,34]. This means that if a study determined that a certain miRNA was upregulated in the tumour tissue, the results cannot be projected on other sample sorts. Additionally, many studies were only performed on one or two chosen cell lines [27,49,78,79]. A lot of the mentioned miRNAs were also found in other cancer types [26] and thus may not be NSCLC-specific.

The roles of numerous miRNAs are still unclear and need to be further investigated. Some miRNAs are stipulated to act both as oncomirs and as tumour suppressors [61]. Other miRNAs were shown to restrain the efficiency of some treatment methods, but support the tumour inhibiting properties of others. Various questions arise in the case of such miRNAs. Does one of the roles outweight the other? Could it still be beneficial to down or upregulate said miRNA, or is it best to keep its levels in line with the results obtained from healthy control groups? Would it be possible to prevent the negative effects of the said double-acting miRNA with the use of any known drugs or alternatively to develop a new method to selectively inhibit its pro-tumorigenic function without the loss of the anti-tumorigenic function?

Some data indicates that different diagnostic panels might be required for male and female patients, as well as for smokers and non-smokers [39]. Many miRNAs were found to be specific to certain tumour types [39,49], which would also make them a suboptimal choice for early NSCLC detection—for example, the miR-21, miR-26, and miR-29a [39] expression changes were different in the AC than in the SCC cases. For the purposes of early NSCLC detection, an optimal diagnostic panel should be able to detect all the NSCLC subtypes at once. Alternatively, a few different NSCLC subtype-specific panels would have to be used, however, this solution seems to be suboptimal, due to the increased costs of such a strategy. Additionally, such a panel would have to detect the NSCLC in all stages, which brings up another issue. Many studies focus only on patients with a certain disease stage [7,39]. However, numerous stage-related differences in the miRNAs’ (e.g., miR-25, miR-30a, miR-126, miR-154) expressions were found [39,41,44,69,80]. For that reason, the results obtained in a trial involving patients with a specific disease stage cannot be projected on other disease stages. A good NSCLC detection panel should also include miRNAs that are specific to it.

It is also important to acknowledge that the results obtained by some researchers could be influenced by the relatively small sample sizes.

Last, but not least, it is possible, that other, to this point, unknown factors are involved in the tumorigenesis, and that they do affect the research results.

## 5. Conclusions

The evidence indicates that miRNAs could be utilised as diagnostic markers and prognostic tools in patients with NSCLC. It also seems that there are many ways in which miRNAs could be used to improve the effectiveness of the currently available NSCLC therapies or even as therapeutic agents themselves. However, the current gaps in knowledge are large and more research is required for better understanding of the role that miRNAs play in the development of the NSCLC.

## Figures and Tables

**Table 1:** The prognostic role of the miR-21 in NSCLC.

Paper Type, Materials and Methods	Expression Changes and Their Relevance for the Clinical Outcome	Authors and Year of Publication
Literature review [40].	↓ expression in the exosomes derived from human bronchial epithelium (HBE) resulted in inhibited angiogenesis. ↑ expression could be correlated with the brain metastases development in the NSCLC patients [40].	Tirpe et al., 2020 [40]
Research article. The miRNAs’ expression was analysed in 165 AC and 125 SCC tissue samples obtained from EAGLE (Environment and Genetics in Lung Cancer Etiology). The utilised tissues were retrieved from the NSCLC patients in years 2003–2005 [39].	↑ expression in stage II AC compared to the SCC. Unrelated to smoking frequency in AC patients and related to it in the SCC patients. Could be a marker of tumour progression in AC [39].	Landi et al., 2010 [39]
Research article. The miRNAs’ expression profiles were measured in tumour-derived exosomes retrieved from 46 stage I NSCLC patients. Additionally, 42 healthy subjects were enrolled in the study as a control group [7].	↑ miR-21-5p expression in AC and SCC samples. Low correlation between the values in circulating plasma and in tumour-derived exosomes. No significant correlation between its expression in the tumour-derived and plasma-derived exosomes [7]. It had been reported that miR-21 has a prognostic value in the NSCLC ^1^ [7].	Jin et al., 2017 [7]
Research article. The utilised plasmasamples were collected from 39 NSCLC patients who had received a TKI-EGFR treatment for advanced EGFR-mutated NSCLC with sensitizing mutations. Additionally, the following human NSCLC cell lines were purchased or obtained from other researchers: A549, NCI-H1299, NCI-H23, NCI-H3255, NCI-H1650, HCC-827, HCC-827GR5, PC-9 [42].	↑ expression in the resistant cell lines correlated with an increased Akt phosphorylation. The miR-21 downregulation resulted in the PI3K-AKT (PI3K—phosphatidylinositol 3-kinase) pathway’s inhibition and an increase in the tumour cells’ drug sensitivity. However, the data on its prognostic role was partially inconsistent [42].	Leonetti et al., 2021 [42]

^1^ The cited paper refers to other sources when providing this information, it is not a direct result of the experimental part of the authors’ research. ↑—respectively increased (expression), ↓—respectively decreased (expression).

**Table 2:** The table below shows how the expression of certain miRNAs correlated with the cisplatin-resistance.

The miRNA	Cisplatin-Resistance Change in Relation to an Increase in the miRNAs’ Expression	Utilised Samples and Cell Lines	Action Mechanisms, Targets and Affected Pathways Related to the Cisplatin Resistance.
miR-15	↑ [47]	Human PC9 and A549 cell lines.	GSK-3β/MCL-1 pathway [47].
		Cisplatin resistant PC9 and A549 cells obtained through the exposition of the PC9 and A549 to increasing doses of cisplatin [47].	
miR-25-3p	↑ [46]	A549 and H1299 cell lines.	3’ UTR (untranslated region) of PTEN, PTEN/PI3K/Akt signalling pathway [46].
		Cisplatin-resistant A549 and H1299 cells generated from the A549 and H1299 cells through their exposition to different cisplatin concentrations.	
		Tumour samples retrieved from previously tumour-inoculated mice [46].	
miR-26a	↑ [48,49]	Not applicable [48].	HMGA1-mediated E2F-Akt pathway, EZH2 [48].
		/	/
		Plasma samples from patients with advanced or metastatic NSCLC obtained before and after the start of the first-line platinum-based chemotherapy treatment and from healthy volunteers [49].	NF-κB, MAPK [49].
miR-31	↑ [28]	Not applicable.	ABCB9 [28]
miR-126-5p	↓ [50]	Tumour tissue samples and adjacent normal lung tissue samples collected from the NSCLC patients.	PTEN/PI3K/Akt signaling pathway via ADAM9 [50].
		Normal human bronchial epithelial BESA-2B cells, human lung AC A549 cells and H1650 cells.	
		Cisplatin-resistant A459/DDP and H1650/DDP cells [50].	
miR-146a	↓ [25]	Not applicable.	Cyclin J, ATG12 (autophagy-related protein 12), JNK2 (c-Jun N-terminal kinase), p53 gene, Bcl2 (B cell lymphoma 2)CEACAM6 protein, TNF-α through NF-κB, IRAK1, and TRAF6 [25].
miR-320	↓ [51]	Not applicable.	MAPK signaling pathway, ErbB signaling pathway [51].
miR-494	↑ [52]	A549, 293T, and H460 cell cultures [52].	CASP2, TNF signaling pathways, NF-κB signaling pathway, apoptosis pathway [52].
miR-556-5p	↑ [53]	Cancer tissue and normal tissue samples retrieved from the NSCLC patients previously treated or not treated with cisplatin.	The 3’UTR (untranslated region) of the NLRP3 mRNA,
		Cisplatin-sensitive NSCLC A549 and H1299 cells.	Gasdermin D.
		Cisplatin-resistant NSCLC A549/DDP and H1299/DDP cells obtained through a prolonged low-dose exposition to cisplatin of the parental A549 and H1299 cells [53].	Cleaved Caspase-1, IL-1β, IL-18 [53].
miR-4443	↑ [54]	Tumour-derived exosomes retrieved from the NSCLC patients with varied reactions to the cisplatin treatment.	Transmission of the cisplatin resistance to the non-cisplatin resistant cell lines through the exosomes [54].
		Parental A549 cells and generated from them cisplatin-resistant A549 cells [54].	FSP1 (fibroblast-specific protein 1) through the targeting of the METLL3 gene [54].

**Table 3:** The prognostic role of the let-7 family members in NSCLC.

Paper Type, Materials and Methods	Expression Changes and Their Relevance for the Clinical Outcome	Authors and Year of Publication
Research article. Forty-six stage I NSCLC patients (26 with AC and 20 with SCC), 42 healthy subjects and 60 patients with a NSCLC suspicion were enrolled. Tumour-derived exosomes were isolated from the patients’ plasma [7].	A variety of let-7 family members could be used as prognostic markers in lung cancer ^1^ [7].	Jin et al., 2017, [7]
Research article. The cohort study included 80 NSCLC patients and 30 healthy control group subjects. The patient examinations took place from May 2016 to February 2017. Blood samples were retrieved before patients received chemotherapy, radiotherapy, or surgery [31].	Let-7f (including the let-7f-5p) expression was ↓ in the NSCLC patients, in comparison to the healthy patients. At the same time, ↑ let-7f expression was observed in patients with more malignant tumours [31].	Wang et al., 2020, [31]
Research article. The miRNAs’ expression was analysed in 165 AC and 125 SCC tissue samples obtained from EAGLE (Environment and Genetics in Lung Cancer Etiology). The utilised tissues were retrieved from the NSCLC patients in years 2003–2005 [39].	The let 7 family’s expression was affected by smoking in female AC patients but not in male AC, female SCC and male SCC patients. Let-7e had a strong predictive value in smoking male early-stage SCC patients. ↓ Let-7e expression was linked to poor survival [39]. It had been shown that the let-7 family members’ under-expression constituted poor postoperative survival among the lung cancer patients ^1^ [39].	Landi et al., 2010, [39]
Literature review [40].	↓ Let-7b expression correlated with shorter progression-free survival and overall survival [40].	Tirpe et al., 2020, [40]
Research article. The plasma samples were retrieved from 195 NSCLC patients treated with first-line platinum-based chemotherapy in years 2009–2017. Thirty three healthy individuals were enrolled as a control group [49].	↑ Serum miR-202 expression in patients with advanced NSCLC correlated with worse survival. According to a KM plotter analysis, which included mainly samples from patients in the early NSCLC stages, ↓ miR-202 expression was a negative prognostic factor [49].	Monastirioti et al. 2021, [49]
Research article. Tissue samples were collected from 55 stage I-II and 16 stage III-IV NSCLC patients. A459 and NCI-H23 cells were utilized [105].	↓ MiR-98 expression was observed in cancer tissue samples, compared to the adjacent tissue. ↑ MiR-98 tissue expression correlated with better overall survival—the median survival length was 50 months and 30 months, respectively, for patients with high and for those with low miR-98 expression. MiR-98 targeted the 3′-UTR of TWIST and inhibited its function, thus limiting the TWIST-mediated EMT. Additionally, it down-regulated cell proliferation through the TWIST-Akt-CDK4/CDK6 inhibition and induced cell apoptosis through the activation of the TWIST-Akt-bcl2/Bax pathway [105]. It had also been shown that miR-98 promoted cisplatin sensitivity through the p53 activation and HMGA2 inhibition. Moreover, it downregulated the PAK1 and ITGB3, which also contributed to the inhibition of the cell proliferation [105].	Zhou et al., 2017, [105]

^1^ The cited paper refers to other sources when providing this information, it is not a direct result of the experimental part of the authors’ research.

## Data Availability

Not applicable.
